# Acute effects of ballistic versus heavy-resistance exercises on countermovement jump and rear-hand straight punch performance in amateur boxers

**DOI:** 10.1186/s13102-022-00557-4

**Published:** 2022-08-28

**Authors:** Wenjuan Yi, Chao Chen, Zixiang Zhou, Weijia Cui, Dexin Wang

**Affiliations:** School of Physical Education and Training, Shanghai University of Sport, No. 399 Changhai Road, Shanghai, 200438 China

**Keywords:** Post-activation performance enhancement, Warm-up, Punch impact, Squat jump, Boxing

## Abstract

**Background:**

Ballistic and heavy-resistance exercises may potentially enhance lower body power, which is paramount for the punching performance of amateur boxers. This study aimed to determine the acute effects of ballistic exercise (BE) and heavy-resistance exercise (HRE) on countermovement jump (CMJ) and rear-hand straight punch performance in amateur boxers.

**Methods:**

Ten amateur boxers performed two conditioning exercises in a randomized and counterbalanced order as follows: squat jump with 4 sets × 8 repetitions at 30% one-repetition maximum (1RM) for BE and squat with 3 sets × 5 repetitions at 80% 1RM for HRE. The jump height (JH), relative maximal force (RMF), relative maximal power (RMP) of the CMJ, punch force (PF), and punch speed (PS) of a rear-hand straight punch were measured before and 3, 6, 9, and 12 min after either BE or HRE.

**Results:**

No significant condition × time interaction was found for JH (*p* = 0.303), RMF (*p* = 0.875), RMP (*p* = 0.480), PF (*p* = 0.939), and PS (*p* = 0.939). In addition, no main effect of the condition for JH (*p* = 0.924), RMF (*p* = 0.750), RMP (*p* = 0.631), PF (*p* = 0.678), and PS (*p* = 0.712). A significant main effect of time was observed for PF (*p* = 0.001) and PS (*p* = 0.001), whereas JH (*p* = 0.081), RMF (*p* = 0.141), and RMP (*p* = 0.430) were not. Pairwise comparison identified that PF (*p* = 0.031) and PS (*p* = 0.005) significantly increased at 9 min compared with those at baseline.

**Conclusions:**

The findings of this study demonstrated that BE and HRE protocols can potentiate the rear-hand straight punch performance at 9 min but bring less favorable improvements for JH, RMF, or RMP of CMJ.

## Background

The rear-hand straight punch is a powerful technique for generating heavy impacts and is primarily used when attempting to knock out an opponent [1]. According to Loturco et al. [1], the punching force (PF) can be considered one of the most important indicators for assessing punching performance. However, the legs are the primary source of PF [2]. A previous study reported that 38.6% of PF may be attributed to the lower extremity of experienced boxers [2]. James et al. [3] confirmed that the explosive power generated by the lower body is vital to combat sports. Specifically, the lower limb plays a central role during punches, and the power produced by the legs is paramount to punching performance [1]. Jumping ability is positively correlated with specific fighting techniques, such as punching acceleration in karate and throwing techniques in judo [1, 4]. Essentially, the importance of lower body power for executing the optimal punch performance is highlighted. Overall, these studies consistently indicated that the explosive power of the lower limb is a major determinant of punch performance.

Neuromuscular performance (maximal strength, explosive power, speed, and throwing) can be transiently increased following conditioning contraction, which may be attributed to post-activation performance enhancement (PAPE) [5]. Conditioning contraction refers to the exercise responsible for eliciting PAPE [6]. Heavy-resistance exercise (HRE) and ballistic exercise (BE) are two common methods used to elicit PAPE [7–9]. Typically, HRE involves a maximal or submaximal load, which is ≥ 80% one-repetition maximum (1RM) for dynamic or isometric maximal voluntary contractions [8, 10]. Several studies have proposed that HRE can improve explosive power in the upper [11] and lower limbs [12, 13]. In contrast, Carbone et al. [14] and Zagatto et al. [15] revealed that HRE failed to induce PAPE in rugby and basketball players. Previous studies have suggested that conditioning exercises may elicit fatigue [6]. The occurrence of PAPE can be affected by the balance between fatigue and potentiation [6, 16, 17]. HRE may generate a high level of fatigue [17]; thus, the equivocal findings among the existing studies may be due to HRE-induced fatigue [14]. According to this notion, much research has been conducted on BE, which is characterized by a low load [18]. A growing body of evidence indicates that BE is an effective way to elicit PAPE [7, 8]. West et al. [8] suggested that BE can be applied to improve the peak power output for upper-body, with effects similar to those of HRE. This result is in agreement with those reported by Zagatto et al. [19]. They reported that the drop jump can enhance repeated sprint ability for professional basketball players. Nevertheless, Hester et al. [18] proposed that neither ballistic nor heavy-load exercises can increase the vertical jump performance in resistance-trained men. Although inconsistent results exist, it has been confirmed that HRE and BE protocols can be applied to improve lower limb power [12, 19]. As mentioned earlier, the major determinant of punch performance is the lower body power [2, 3]. Thus, HRE and BE can enhance the lower limb power of amateur boxers and further enhance punch performance. Interestingly, BE involves performing movements with maximal velocity, and the neuromuscular can be activated within a few milliseconds [16]. Maloney et al. [16] reported that the threshold of motor unit recruitment for rapid contraction was lower than that for ramped contraction. This is consistent with Masakado et al. [20] who demonstrated that the threshold of the motor unit decreases with an increase in the speed of muscle contraction. The magnitude of PAPE may be affected by the activation of the motor unit and the type of muscle fibers [6, 16]; therefore, ballistic movements may activate faster motor units and exert a greater PAPE response compared with non-ballistic movements.

The purpose of this study was to investigate the acute effects of BE and HRE on countermovement jump (CMJ) and punch performance in amateur boxers. Additionally, we compared the effects of BE and HRE to determine which type of conditioning exercise yielded greater enhancement for boxers. We hypothesized that 1) both BE and HRE would improve CMJ and rear-hand straight punching performance, and 2) BE would induce a greater PAPE response than HRE.

## Methods

### Experimental approach to the problem

A within-participant repeated-measures design was adopted because each participant completed one familiarization session and two testing sessions. During the familiarization session, anthropometric, 1 repetition maximum (1RM) squats, and medical history were obtained. The familiarization session was conducted at least 96 h before any testing session, and two testing sessions were separated by at least 72 h [18]. Each participant completed two testing sessions at the same time of day (8:00–10:00 a.m.), and in similar ambient conditions (~ 22.5 °C air temperature and ~ 60% of humidity) to avoid any influence of circadian rhythms and diurnal variation [7, 18]. The following two main trials were considered: ballistic exercise (BE) and heavy-resistance exercise (HRE). Our study was conducted based on pre-post testing of jump height (JH), relative maximal force (RMF), relative maximal power (RMP) of CMJ, punch force (PF), and punch speed (PS) of rear-hand straight punch. Additionally, percentage changes were used to quantify the PAPE responses at different time points [21].

### Participants

Ten amateur boxers volunteered to participate in this study, which was approved on September 15, 2021, by the Institutional Review Board of the Shanghai University of Sport (number: 102772021RT102; chairperson: Yan Tang). The physical characteristics of the participants are presented in Table 1. All participants took part in 6 to 8 training sessions per week and had no injuries or diseases, no history of drug abuse, or medications (which are known to affect the neuromuscular system). They were asked to refrain from caffeine for at least 6 h and from strenuous exercise for at least 48 h before testing. All participants had the right to terminate their participation.

**Table 1:** Descriptive statistics of the participants

Variable	Mean ± *SD*	Range
Age (years)	19.20 ± 1.55	18–21
Height (cm)	171.60 ± 5.06	165–182
Body mass (kg)	63.58 ± 6.83	55.3–77.4
Training experience (years)	5.40 ± 2.54	2–9.5
1 RM squat (kg)	90.80 ± 8.39	82–107

1 RM, one repetition maximum

### Procedures

The initial visit to the laboratory was designed to familiarize them with the test protocols by performing a series of squats, squat jumps, CMJ, and rear-hand punches with a 2–3 min recovery. Anthropometric data were recorded during familiarization session. All participants provided written informed consent after a thorough explanation of the testing protocol and possible risks. Additionally, 1RM in the squats of each participant was measured in this session. A standardized warm-up routine, which included 5 min of self-paced jogging on a treadmill and 5 min of dynamic stretching, was used for each preliminary and experimental test.

**Fig. 1:**
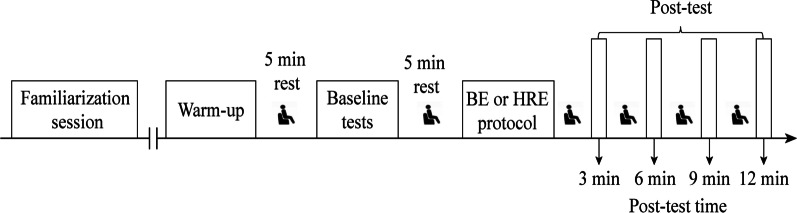
Experimental procedures. BE = ballistic exercise; HRE = heavy-resistance exercise

A 5 min rest was allowed before the baseline measurement of each variation (CMJ and rear-hand straight punch). The participants subsequently performed the BE or HRE protocol 5 min after baseline measurements. The two PAPE protocols were completed in a randomized fashion. The BE protocol consisted of 4 sets of 8 repetitions of squat jumps loaded with 30% 1RM [8], whereas the HRE protocol consisted of 3 sets of 5 repetitions of squats loaded with 80% 1RM. It has been established that a 30% 1RM squat jump and 80% 1RM squat can be used to transiently improve lower body explosive power [10, 22], and multiple-set conditioning exercises can induce a greater PAPE response than single-set exercises [17]. However, to date, there is a lack of consensus regarding the repetition of conditioning exercises. For both BE and HRE protocols, there was 90 s of passive recovery between sets [18]. Following BE and HRE protocols at 3, 6, 9, and 12 min [10, 16], the participants performed the CMJ and rear-hand straight punch three times for each time delay. The best attempts among each triplet were considered for analysis. The experimental procedure is illustrated in Fig. 1.

### Measurements

#### Strength testing

Muscular strength (1RM) was evaluated for squat exercises following a self-paced warm-up routine. The participants performed two sets of warm-up exercises, consisting of 10 repetitions at 50% 1RM and 5 repetitions at 75% 1RM, which were estimated according to their training logs [23]. The participants were allowed to rest for 3 min to recover between sets. They were subsequently asked to perform a repetitions test to evaluate their present squat 1RM. This repetitions test allowed the participants to complete 4–8 repetitions before failing. The 4–8 repetitions corresponded to 80–90% 1RM, which has been applied to predict 1RM [23]. The reasons for applying a prediction test in this study were to reduce the risk of injury for participants; moreover, the 1RM can be accurately predicted based on the measured repetitions [23]. Squat depth was judged at the point when the thigh was considered to be in a “parallel” position where the greater trochanter of the femur was aligned with the knee [24]. Visual inspection was conducted by a researcher to ensure that the participants reached the desired depth, who also provided verbal instruction of “UP” to indicate that the desired depth had been achieved [25].

#### Measurement of CMJ performance

CMJ was measured using a force platform (Kistler, Winterthur, Switzerland; 900 × 600 mm; model 9287B) and the sampling frequency was set at 1000 Hz. The data were low-pass filtered with a 20 Hz cut-off frequency and exported from the BioWare software (BioWare version 5.3.0.7; Kistler, Winterthur, Switzerland) for further analysis [26]. The participants were instructed to stand on the force platform in an upright position and remain still for at least 2 s to measure their body weight. They were then instructed to dip to a self-selected depth prior to jumping vertically as explosively as possible with their hands fixed at their hips at all times [24]. CMJ testing was monitored by an experienced operator, and vocal encouragement was provided to the participants. The following variables were analyzed in this study: JH (m), RMF (%BW) and RMP (W/kg) [24]. JH was calculated from the “flight” time in the air between take-off and landing; RMF was the maximal force normalized to the participant’s body weight; RMP was the maximal power normalized to the participant’s body weight [24].

#### Measurement of rear-hand straight punching performance

The participants were allowed 5 min (or longer, if necessary) to familiarize themselves with and adjust the height of the punching bag. The punching bag (Wesing, Fujian, China) was made of cowhide leather, filled with high-density foam, and safely secured to a wall bracket using a stainless-steel heavy bag chain. The participants wore 10-oz competition boxing gloves (Wesing, Fujian, China) over regular fabric hand wraps (length 450 cm, width 5 cm; Wesing, Fujian, China) and were instructed to assume an orthodox stance to perform a rear-hand straight punch. All the participants were required to perform a single maximum-effort rear-hand straight punch [1]. For each attempt, two researchers provided supervision to guarantee the proper technique of punches and vocally encourage athletes. A professional boxing transducer (Strike Tec Boxing Sensors, Strike Tec, Dallas, USA; version 1.4.4) with a custom-designed mobile application was used to evaluate PF (N) and PS (m/s). The data of PF and PS were calculated from acceleration and can be extracted from the mobile application [27]. It has been reported that there is high reliability for PF (typical error of measurement: 0.57) [28] and PS (ICC: 0.853; 95% confidence intervals: 0.650–0.942) [29] when calculated from acceleration.

### Statistical analyses

Descriptive data were presented as mean and standard deviations (SD). Normality was determined using the Shapiro–Wilk test and Levene’s test for examining the homogeneity of variance. Intraclass correlation coefficients (ICC) were calculated by correlating the baseline values (i.e., JH, RMF, RMP, PF, and PS) in two experimental sessions. Two-way (2 × 5 [conditions × times]) repeated measures analysis of variance was used to determine whether significant differences existed between conditions for JH, RMF, RMP, PF, and PS at five time points (i.e., baseline and 3, 6, 9, and 12 min after the PAPE protocols). Mauchley’s test was used to verify the sphericity for each analysis. When sphericity inconformity was identified, a Greenhouse–Geisser adjustment was applied. Pairwise comparisons with Bonferroni correction were performed if any significant differences occurred. The level of statistical significance was set at *p* ≤ 0.05. All statistical analyses were conducted using IBM SPSS for Windows version 25.0 (SPSS, Inc., Chicago, IL, USA).

## Results

**Fig. 2:**
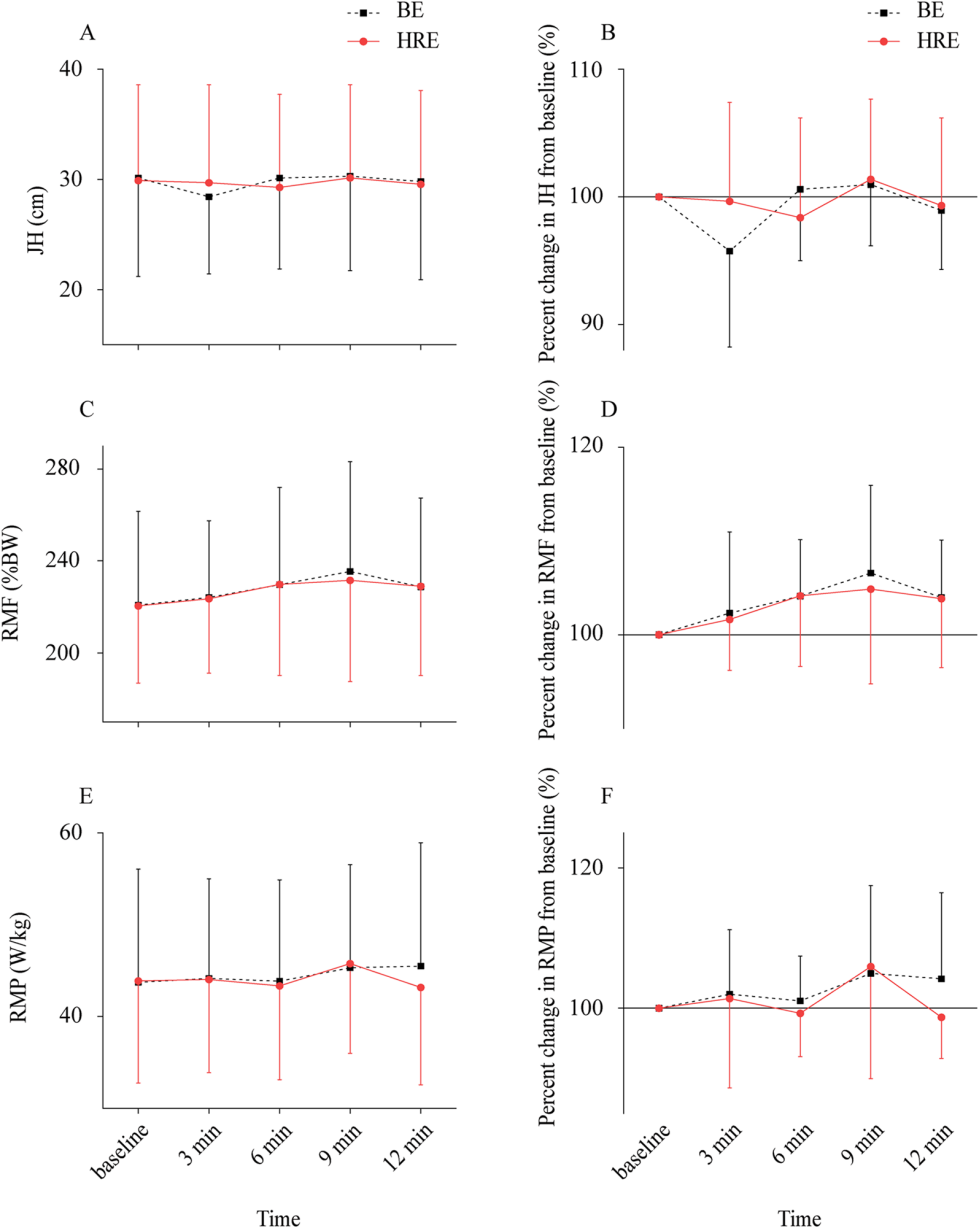
Absolute values and percent change for JH, RMF, and RMP (Mean ± SD). Absolute values for JH (**A**), percent change for JH (**B**), absolute values for RMF (**C**), percent change for RMF (**D**), absolute values for RMP (**E**), percent change for RMP (**F**). JH = jump height; RMF = relative maximal force; RMP = relative maximal power; BE = ballistic exercise; HRE = heavy-resistance exercise

**Fig. 3:**
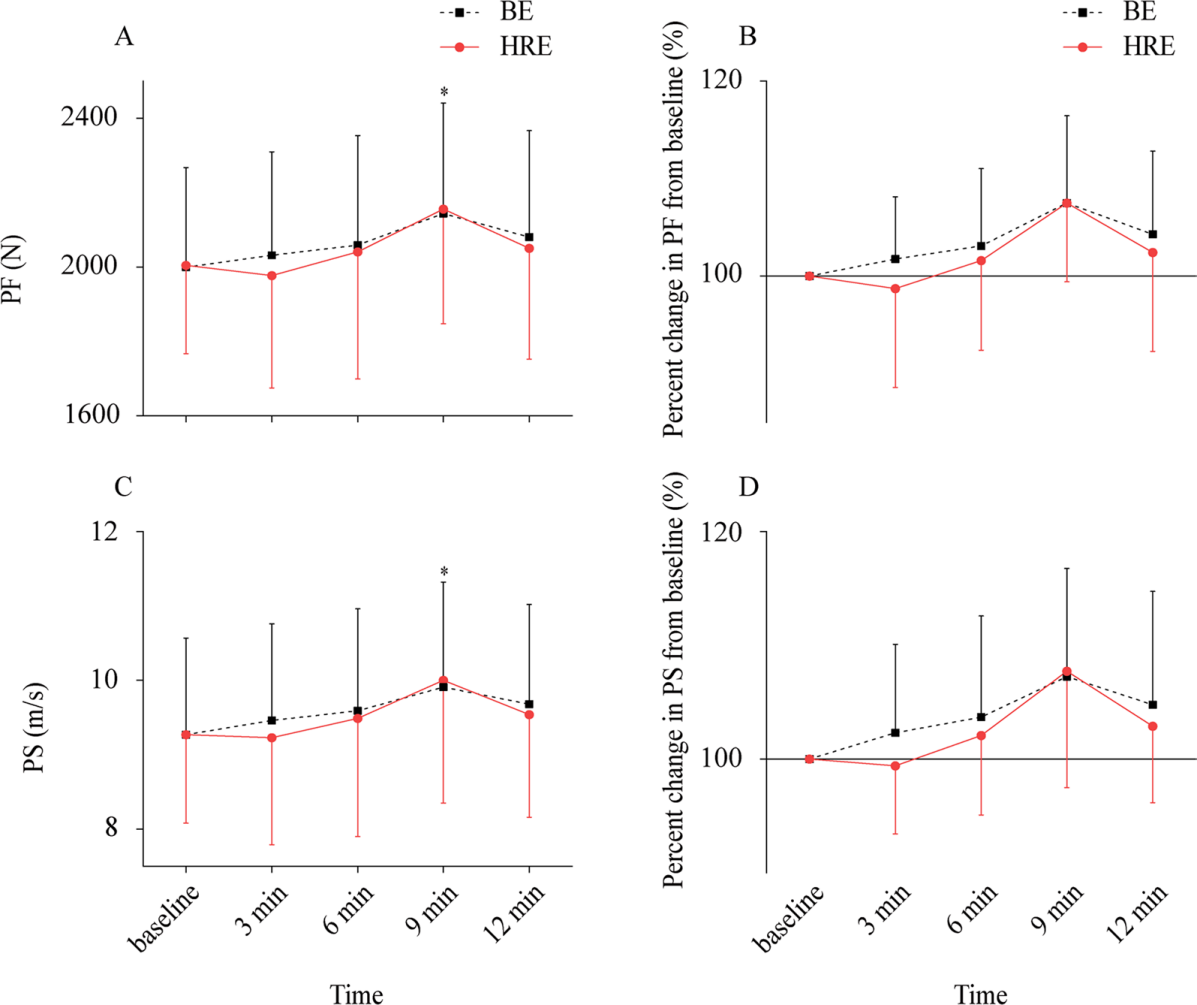
Absolute values and percent change for PF and PS (Mean ± SD). Absolute values for PF (**A**), percent change for PF (**B**), absolute values for PS (**C**), percent change for PS (**D**). PF = punch force; PS = punch speed; BE = ballistic exercise; HRE = heavy-resistance exercise. ^*^ = significant differences to baseline (*p* ≤ 0.05)

Statistical analysis revealed that all data were normally distributed (*p* > 0.05) and conformed to the homogeneity of variance (*p* > 0.05). The ICC for JH, RMF, RMP, PF, and PS were 0.972, 0.962, 0.986, 0.903, and 0.912, respectively. No significant condition × time interaction was noted for JH (*p* = 0.303), RMF (*p* = 0.875), RMP (*p* = 0.480), PF (*p* = 0.939), and PS (*p* = 0.939). No main effect of the condition was observed for JH (*p* = 0.924), RMF (*p* = 0.750), RMP (*p* = 0.631), PF (*p* = 0.678), and PS (*p* = 0.712). However, a significant main effect of time was observed for PF (*p* = 0.001) and PS (*p* = 0.001), whereas JH (*p* = 0.081), RMF (*p* = 0.141), and RMP (*p* = 0.430) were not. Compared with the baseline, pairwise comparisons identified significance at 9 min after conditioning exercises for PF (*p* = 0.031) and PS (*p* = 0.005). In comparison with the baseline, the values in PF (BE: 2143.46 ± 296.92 N [7.49% ± 8.94%], HRE: 2155.62 ± 308.25 N [7.49% ± 8.09%]) and PS (BE: 9.91 ± 1.41 m/s [7.25% ± 9.51%]), HRE: 10.0 ± 1.65 m/s [7.73% ± 10.25%]) revealed the punching performance significantly increased at 9 min. The absolute values and percent changes for CMJ and rear-hand straight punching performance across all time points for each conditioning exercise are presented in Figs. 2 and 3.

## Discussion

The present study aimed to determine whether BE and HRE protocols were designed to augment CMJ and rear-hand straight punch performance in amateur boxers. The results of the current study partially support the first hypothesis that both BE and HRE protocols would significantly improve CMJ and rear-hand straight punch performance. However, the main findings of this study revealed that except for PF and PS, which significantly increased at 9 min, the variables of CMJ were not different from the baseline values for either protocol. Furthermore, the results of this study do not support the second hypothesis that BE exerts greater benefits than HRE. The results showed that PF and PS increased, with no difference between conditioning exercises being reported.

To date, a large number of studies have demonstrated that the explosive power of the lower limb can be transiently enhanced by conditioning exercises [18, 23]. BE and HRE have been established as modalities for exploiting PAPE; however, there is a lack of consensus on the effects of these two exercises [14]. In this study, BE and HRE were able to remarkably increase punching performance (i.e., PF and PS) at 9 min compared with those at baseline. The results of this study were comparable with those of Terzis et al. [30] and Dolan et al. [31] who applied conditioning exercises in lower body to improve upper body performance. More specifically, Terzis et al. [30] used a drop jump to potentiate throwing performance and found a significant improvement in men. Dolan et al. [31] suggested that the hang clean and jerk can also augment shot put throw performance in track and field athletes. However, it should be noted that BE and HRE failed to increase the JH, RMF, and RMP of CMJ in the present study. This finding of the present study was congruent with previous literature [32], indicating that neither ballistic nor heavy-load exercises improve CMJ performance in combat sports athletes. The results obtained by Hanson et al. [23] were contradictory, and they proposed that both light- and heavy-load squats can enhance jump performance. It is important to note that a greater PAPE response can be realized among those with more resistance training experience [17]. The participants of Hanson et al. [23] were resistance-trained male and female, thereby the discrepancies between the results of the present study and those of Hanson et al. [23] may be due to the resistance training status or/and strength level of the participants being different. Furthermore, differences in the type and load of the conditioning exercises can also bring about inconsistent results. It has been well established that the explosive power of the lower limbs makes a considerable contribution to the punch performance [2]. Although the variables of CMJ (i.e., JH, RMF, and RMP) did not significantly change after BE or HRE, our findings revealed 0.96–1.37% (30.29 ± 8.57 cm vs. 30.14 ± 8.46 cm), 4.85–6.57% (235.40 ± 47.84%BW vs. 231.51 ± 44.0%BW), 4.96–5.95% (45.30 ± 11.24 W/kg vs. 45.75 ± 9.77 W/kg) improvements in JH, RMF, and RMP at 9 min. The improvements in CMJ can be due to the muscle of the lower limb activated by conditioning exercises; however, it is insufficient to elicit the degree of potentiation required to distinctly differentiate from baseline [16, 18]. Moreover, it is plausible that PF and PS may benefit from the muscular activation of the lower limbs. Boxing requires the leg, trunk, and arm to follow a correct kinetic chain to perform optimal punches [33]. Although the explosive power of the lower body is paramount to punch performance, boxing emphasizes the transmission of power from the lower body to the arm [33]. The participants in this study participated in boxing training regularly, and they had built specific muscular coordination. Miarka et al. [34] proposed that athletic performance may be influenced by the integration of muscular coordination. Thus, lower body conditioning exercises may be reflected in the built-in muscular coordination of boxers, that is, BE and HRE can only potentiate punching performance.

It has been suggested that BE can activate type II muscle fibers and generate greater power output owing to high level of acceleration throughout the range of motion [16, 20]. In contrast, HRE contains a deceleration phase that may affect movement velocity, muscle activation, and power production [16]. Moreover, light-load exercises may produce less fatigue than heavy-load exercises [17]. Consequently, BE may be more effective than HRE in inducing PAPE. Nevertheless, the punching performance of the amateur boxers was equally improved by BE and HRE in this study. This improvement in punching performance is comparable to the increase demonstrated in previous literature, which also used ballistic [30, 35] and heavy-load exercises [31] as PAPE protocols. For example, Karampatsos et al. [35] reported an increase in throwing performance in track and field throwers, when using a conditioning exercise with 3 consecutive CMJs. Terzis et al. [30] and Dolan et al. [31] proposed that throwing performance can be increased after conducting 5 maximal consecutive drop jumps or 3 repetitions of a hang clean and jerk at 80% 1RM. The aforementioned studies demonstrate that BE can realize PAPE to a similar degree as HRE. The mechanisms of the comparable potentiation effects between BE and HRE are possibly due to the maximal recruitment of the motor units [8]. Prior research suggests that heavy-load exercises can elicit PAPE due to the higher recruitment of motor units comprising type II muscle fibers than light-load exercises [16]. The explosive nature of BE can activate neuromuscular activity within a few milliseconds, thereby generating greater power output [8, 16]. Therefore, theoretically, this can illustrate the reason why HRE and BE could equally increase PF and PS for amateur boxers in this study.

Based on the results of the current study, BE and HRE can be considered part of the warm-up routine for enhancing punch performance during competition or aiming to develop explosive power in training. However, PAPE occurred 9 min after the conditioning exercise for amateur boxers, thereby requiring optimal recovery time between the conditioning exercise and the subsequent exercise. Although the BE and HRE were unable to exert a PAPE response for the lower limbs, muscle activation of the lower limb may contribute to an increase in the punching performance.

## Limitations

Some limitations should be considered in this study. First, although we attempted to reduce the risk of testing, potential risks still existed. For example, participants had to perform maximal CMJs and rear-hand punches. Second, special attention should be paid to the results of CMJ, which may be negatively affected by the learned movement structure. The participants in this study were amateur boxers who were proficient in punching and not in CMJ. Third, the testing of CMJ and punching performance in different recovery intervals may act as conditioning exercises; therefore, it would exert a potential effect on the PAPE response. A control condition without any additional conditioning contractions may be needed to compare with BE and HRE at different recovery intervals.

## Conclusions

This study suggests that punching performance (i.e., PF and PS) can be improved at 9 min after BE and HRE protocols for amateur boxers, whereas neither BE nor HRE significantly enhances CMJ performance at any recovery interval. Moreover, no distinct gap was noted between the BE and HRE in terms of increasing the punching performance.

## Data Availability

The datasets used and/or analysed during the current study are available from the corresponding author on reasonable request.

## Abbreviations

BE: Ballistic exercise
HRE: Heavy-resistance exercise
CMJ: Countermovement jump
1 RM: One repetition maximum
JH: Jump height
RMF: Relative maximal force
RMP: Relative maximal power
PF: Punch force
PS: Punch speed
PAPE: Post-activation performance enhancement
ICC: Intraclass correlation coefficient
SD: Standard deviation
SPSS: Statistical package of social sciences

## References

[1] Loturco I, Nakamura FY, Artioli GG, Kobal R, Kitamura K, Cal Abad CC (2016). Strength and power qualities are highly associated with punching impact in elite amateur boxers. J Strength Cond Res.

[2] Filimonov VI, Koptsev KN, Husyanov ZM, Nazarov SS (1985). Boxing: means of increasing strength of the punch. J Strength Cond Res.

[3] James LP, Connick M, Haff GG, Kelly VG, Beckman EM (2020). The countermovement jump mechanics of mixed martial arts competitors. J Strength Cond Res.

[4] Zaggelidis G, Lazaridis S (2013). Muscle activation profiles of lower extremities in different throwing techniques and in jumping performance in elite and novice Greek Judo Athletes. J Hum Kinet.

[5] Prieske O, Behrens M, Chaabene H, Granacher U, Maffiuletti NA (2020). Time to differentiate postactivation "potentiation" from "performance enhancement" in the strength and conditioning community. Sports Med.

[6] Tillin NA, Bishop D (2009). Factors modulating post-activation potentiation and its effect on performance of subsequent explosive activities. Sports Med.

[7] Gil MH, Neiva HP, Garrido ND, Aidar FJ, Cirilo-Sousa MS, Marques MC (2019). The effect of ballistic exercise as pre-activation for 100 m sprints. Int J Environ Res Public Health.

[8] West DJ, Cunningham DJ, Crewther BT, Cook CJ, Kilduff LP (2013). Influence of ballistic bench press on upper body power output in professional rugby players. J Strength Cond Res.

[9] Izquierdo SM, Bautista IJ, Martin F (2020). Post-activation performance enhancement (PAPE) after a single-bout of high-intensity flywheel resistance training. Biol Sport.

[10] Dobbs WC, Tolusso DV, Fedewa MV, Esco MR (2019). Effect of postactivation potentiation on explosive vertical jump: a systematic review and meta-analysis. J Strength Cond Res.

[11] Tsoukos A, Brown LE, Veligekas P, Terzis G, Bogdanis GC (2019). Postactivation potentiation of bench press throw performance using velocity-based conditioning protocols with low and moderate loads. J Hum Kinet.

[12] Mangine GT, Ratamess NA, Hoffman JR, Faigenbaum AD, Kang J, Chilakos A (2008). The effects of combined ballistic and heavy resistance training on maximal lower- and upper-body strength in recreationally trained men. J Strength Cond Res.

[13] Kumar SS, Shahid R, Ali MJ, Shalini V, Husain NI, Shahnawaz A (2018). Postactivation potentiation following acute bouts of plyometric versus heavy-resistance exercise in collegiate soccer players. Biomed Res Int.

[14] Carbone L, Duarte MG, Chulvi-Medrano I, Bonilla DA, Alexandre AA (2020). Effects of heavy barbell hip thrust vs back squat on subsequent sprint performance in rugby players. Biol Sport.

[15] Zagatto AM, Claus GM, Dutra YM, de Poli RA, Lopes VHF, Goodall S (2022). Drop jumps versus sled towing and their effects on repeated sprint ability in young basketball players. BMC Sports Sci Med Rehabil.

[16] Maloney SJ, Turner AN, Fletcher IM (2014). Ballistic exercise as a pre-activation stimulus: a review of the literature and practical applications. Sports Med.

[17] Seitz LB, Haff GG (2016). Factors modulating post-activation potentiation of jump, sprint, throw, and upper-body ballistic performances: a systematic review with meta-analysis. Sports Med.

[18] Hester GM, Pope ZK, Sellers JH, Thiele RM, DeFreitas JM (2017). Potentiation: effect of ballistic and heavy exercise on vertical jump performance. J Strength Cond Res.

[19] Zagatto AM, Dutra YM, Claus G, Malta E, Boullosa DA (2022). Drop jumps improve repeated sprint ability performance in professional basketball players. Biol Sport.

[20] Masakado Y (1995). Motor unit firing behavior in slow and fast contractions of the first dorsal interosseous muscle of healthy men. Electroencephalogr Clin Neurophysiol.

[21] Cleary CJ, Cook SB (2020). Postactivation potentiation in blood flow-restricted complex training. J Strength Cond Res.

[22] Suchomel TJ, Lamont HS, Moir GL (2015). Understanding vertical jump potentiation: a deterministic model. Sports Med.

[23] Hanson ED, Leigh S, Mynark RG (2007). Acute effects of heavy- and light-load squat exercise on the kinetic measures of vertical jumping. J Strength Cond Res.

[24] Mitchell CJ, Sale DG (2011). Enhancement of jump performance after a 5-RM squat is associated with postactivation potentiation. Eur J Appl Physiol.

[25] Nickerson BS, Williams TD, Snarr RL, Park K-S (2019). Individual and combined effect of inter-repetition rest and elastic bands on jumping potentiation in resistance-trained men. J Strength Cond Res.

[26] Tsoukos A, Bogdanis GC, Terzis G (2016). Acute improvement of vertical jump performance after isometric squats depends on knee angle and vertical jumping ability. J Strength Cond Res.

[27] Omcirk D, Vetrovsky T, Padecky J, Vanbelle S, Malecek J, Tufano JJJS (2021). Punch trackers: correct recognition depends on punch type and training experience. Sensors.

[28] Lenetsky S, Brughelli M, Nates RJ, Cross MR, Lormier AV (2018). Variability and reliability of punching impact kinetics in untrained participants and experienced boxers. J Strength Cond Res.

[29] Harris DM, Caillaud K, Khullar S, Haff GG, Latella C (2021). The reliability of a linear position transducer and commercially available accelerometer to measure punching velocity in junior boxing athletes. Int J Sports Sci Coach.

[30] Terzis G, Spengos K, Karampatsos G, Manta P, Georgiadis G (2009). Acute effect of drop jumping on throwing performance. J Strength Cond Res.

[31] Dolan M, Sevene TG, Berninig J, Harris C, Climstein M, Adams KJ (2017). Post-activation potentiation and the shot put throw. Int J Sports Sci.

[32] Tsolakis C, Bogdanis GC, Nikolaou A, Zacharogiannis E (2011). Influence of type of muscle contraction and gender on postactivation potentiation of upper and lower limb explosive performance in elite fencers. J Sports Sci Med.

[33] Stanley E, Thomson E, Smith G, Lamb KL (2018). An analysis of the three-dimensional kinetics and kinematics of maximal effort punches among amateur boxers. Int J Perform Anal Sport.

[34] Miarka B, Del Vecchio FB, Franchini E (2011). Acute effects and postactivation potentiation in the Special Judo Fitness Test. J Strength Cond Res.

[35] Karampatsos GP, Korfiatis PG, Zaras ND, Georgiadis GV, Terzis GD (2017). Acute effect of countermovement jumping on throwing performance in track and field athletes during competition. J Strength Cond Res.

